# Medication-related osteonecrosis of the upper jaw involving the zygomatic bone: A case report

**DOI:** 10.1016/j.ijscr.2023.107932

**Published:** 2023-02-17

**Authors:** Mitsunobu Otsuru, Sakiko Soutome, Saki Hayashida, Kota Morishita, Souichi Yanamoto, Masahiro Umeda

**Affiliations:** a Department of Clinical Oral Oncology, Nagasaki University Graduate School of Biomedical Sciences, 1-7-1 Sakamoto, Nagasaki-shi, Nagasaki-ken 852-8588, Japan; b Department of Oral Health, Nagasaki University Graduate School of Biomedical Sciences, 1-7-1 Sakamoto, Nagasaki-shi, Nagasaki-ken 852-8588, Japan

**Keywords:** Osteonecrosis, Skull base, Bisphosphonates, Maxilla, Zygoma, Osteolysis, Case report

## Abstract

**Introduction and importance:**

Osteonecrosis of the jaw resulting from treatment with antiresorptive agents, such as bisphosphonates and denosumab, is widely recognized as medication-related osteonecrosis of the jaw (MRONJ). However, to the best of our knowledge, there are no reports of medication-related osteonecrosis of the upper jaw extending to the zygomatic bone.

**Case presentation:**

An 81-year-old woman treated with denosumab for multiple lung cancer bone metastases presented to the authors' hospital with a swelling in the upper jaw. Computed tomography showed osteolysis, periosteal reaction of the maxillary bone, maxillary sinusitis, and osteosclerosis of the zygomatic bone. The patient underwent conservative treatment; however, osteosclerosis of the zygomatic bone progressed to osteolysis.

**Clinical discussion:**

If the maxillary MRONJ extends to the surrounding bony structures, such as the orbit and skull base, serious complications may occur.

**Conclusion:**

It is important to detect early signs of maxillary MRONJ, before it involves the surrounding bones.

## Highlights

• Treatment with antiresorptive agents may cause osteonecrosis of the jaw.
• Extension of maxillary MRONJ to the zygomatic bone has not been reported.
• Extension of maxillary MRONJ to the adjacent bones may cause serious complications.
• Conservative treatment is the first-line therapy for MRONJ.
• Surgery is recommended in case of poor treatment outcomes.

## 1 Introduction

Since Marx [1] first reported bisphosphonate-induced osteonecrosis of the jaw in 2003, osteonecrosis of the jaw resulting from antiresorptive agent administration has been recognized worldwide as medication-related osteonecrosis of the jaw (MRONJ) [2], [3]. Currently, there are no reports of maxillary MRONJ spreading to the zygomatic bone. The pathological significance of MRONJ involving the surrounding bones has not been widely discussed, probably due to the small number of cases. Herein, we report a unique case of maxillary MRONJ spreading to the zygomatic bone and discuss the pathological significance of MRONJ progression beyond the jawbone to the surrounding facial bones [4].

## 2 Presentation of case

**Fig. 1:**
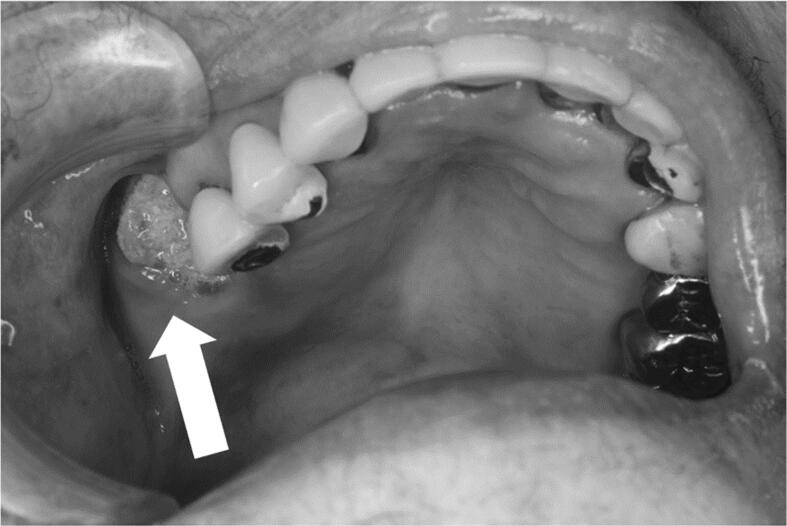
Clinical photograph shows a swelling in the right maxilla and bone exposure with pain in the maxillary right first molar extraction socket (arrow).

**Fig. 2:**
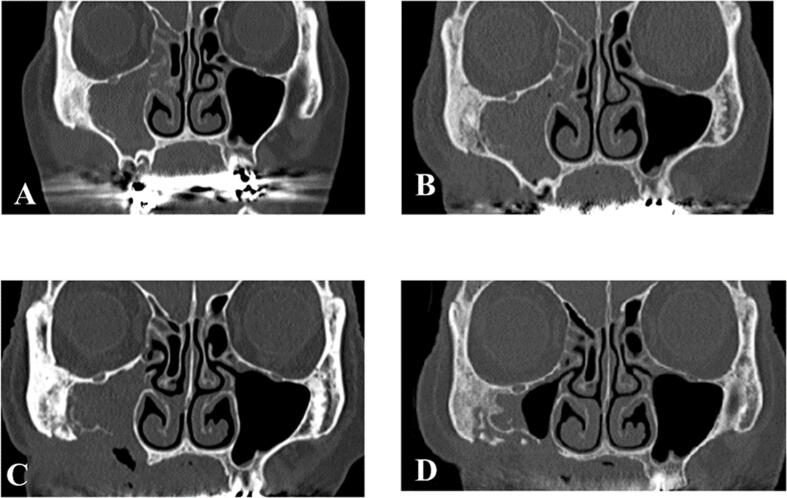
Computed tomography findings (coronal sections). A: Initial visit. B: 1 month later. C: 12 months later. D: 24 months later.

An 81-year-old woman was referred to the Department of Oral and Maxillofacial Surgery at our hospital for a swelling in the upper jaw. The patient had a history of radiation therapy for lung cancer and had been receiving denosumab for >4 years for the treatment of multiple bone metastases. Clinical examination revealed a swelling in the right maxilla and bone exposure with pain in the maxillary right first molar extraction socket (Fig. 1). Computed tomography (CT) showed osteolysis of the right maxilla with maxillary sinusitis. In addition, a periosteal reaction in the posterior wall of the maxillary sinus and osteosclerosis of the zygomatic bone were observed (Fig. 2A). The patient was diagnosed with stage 3 MRONJ. She underwent 6 months of conservative treatment, including rinsing with an antimicrobial agent, administration of antibiotics, and lavage of the socket. Despite treatment, osteolysis of the maxillary bone progressed, and fistula formation was observed in the cheek (Fig. 2B). Therefore, resection of the bony sequestrum and debridement of the fistula were performed under general anesthesia. Thereafter, the painful bone exposure improved, but the cutaneous fistula persisted. Histopathological examination revealed sequestration with granulation tissue and abscess formation. Bacterial culture of the fistular secretions grew methicillin-resistant *Staphylococcus aureus* (MRSA). Eight months after surgery, CT showed progression of osteosclerosis of the zygomatic bone to osteolysis (Fig. 2C). Fifteen months postoperatively, CT revealed separation of the collapsed bone and improvement of the maxillary sinus mucosal thickening (Fig. 2D). Therefore, we diagnosed this case as MRONJ progression rather than a tumor. Although we had planned another surgery 2 years after the first one, the patient was admitted to another hospital for a femoral fracture and was lost to follow-up.

## 3 Discussion

Osteoradionecrosis occurs at the site of exposure to radiation and can appear in the zygomatic bone and skull base; fatal cases have been reported [5], [6]. However, to the best of our knowledge, there are no reports of MRONJ spreading to the zygomatic bone or skull base. Osteoradionecrosis begins at the site of irradiation, whereas MRONJ starts in the jaw before spreading to the adjacent bones. Therefore, it takes longer for symptoms to appear and early detection may be difficult.

MRONJ most commonly occurs in the mandible, and mandibular MRONJ has worse outcomes compared to MRONJ of the maxilla. MRONJ of the maxilla likely has better treatment outcomes due to the rich blood supply of the maxilla. However, the upper jaw has a complicated structure, and it surrounds important organs such as the eyes and brain. Therefore, if maxillary MRONJ extends to the surrounding bony structures, such as the orbit and skull base, serious complications may occur. Thus, early detection of maxillary MRONJ, before surrounding bones are involved, is crucial for favorable outcomes.

In this case, osteosclerosis of the zygomatic bone was observed at the initial visit. To determine the frequency of osteosclerosis, the authors retrospectively examined the surrounding bones of 56 patients diagnosed with maxillary MRONJ, treated between 2010 and 2020 at the Department of Oral and Maxillofacial Surgery, Nagasaki University Hospital. Osteosclerosis of surrounding bones was observed in nine patients, including the current case (involvement of the zygomatic bone in seven patients and the zygomatic bone and pterygoid process of the sphenoid bone in two patients) (Table 1). Of these nine patients, a characteristic periosteal reaction was observed in eight cases, and maxillary sinusitis was observed in all nine cases. Therefore, periosteal reaction and maxillary sinusitis in maxillary MRONJ may be the risk factors for osteonecrosis spreading to the surrounding bones.

**Table 1:** Cases of maxillary MRONJ with osteosclerosis in the surrounding bones.

Case	Sex	Age (Years)	Primary disease	Antiresorptive agent	Administration period	Corticosteroid	Diabetes	Periosteal reaction	Maxillary sinusitis	Surrounding bone involvement
1	Female	60	Malignant tumor	Bisphosphonate	≧4 years	−	−	+	+	Zygomatic bone
2	Female	75	Malignant tumor	Denosumab	<4 years	−	−	+	+	Zygomatic bone
3	Male	63	Malignant tumor	Bisphosphonate	<4 years	−	−	+	+	Zygomatic bone/pterygoid process
4	Male	77	Malignant tumor	Bisphosphonate	<4 years	−	+	−	+	Zygomatic bone/pterygoid process
5	Female	82	Osteoporosis	Bisphosphonate	≧4 years	−	−	+	+	Zygomatic bone
6	Female	79	Osteoporosis	Bisphosphonate	≧4 years	−	−	+	+	Zygomatic bone
7	Female	86	Osteoporosis	Bisphosphonate	≧4 years	−	−	+	+	Zygomatic bone
8	Male	64	Malignant tumor	Bisphosphonate	<4 years	−	+	+	+	Zygomatic bone
9^a^	Female	81	Malignant tumor	Denosumab	≧4 years	−	−	+	+	Zygomatic bone

MRONJ: medication-related osteonecrosis of the jaw.

a The current case.

The organisms isolated from MRONJ are usually bacteria indigenous to the oral cavity, such as *Actinomyces* [7]. However, in this case, MRSA was detected in the fistula, probably due to the intermittent and prolonged use of antibacterial agents. Long-term antibiotic administration may promote the development of resistant strains. Case have also been reported in which MRONJ progressed after prolonged conservative treatment and required extended resection [8]. A position paper of the American Association of Oral and Maxillofacial Surgeons (AAOMS) reported in 2022 also included surgery as an option for MRONJ treatment [9]. In addition, surgery for MRONJ has also been performed in the early stages of the disease [9], [10]. Therefore, early surgical treatment can be considered in special cases, such as maxillary MRONJ with periosteal reaction and maxillary sinusitis, to ensure complete healing and prevent progression of osteonecrosis to the surrounding bones and serious complications.

In conclusion, if maxillary MRONJ extends to the surrounding bones complications may arise and treatment may become challenging. Therefore, it is important to detect early signs of maxillary MRONJ.

## 4 Conclusion

It is important to detect early signs of maxillary MRONJ, before it involves the surrounding bones.

## Patient consent

Written informed consent was obtained from the patient for publication of this case report and accompanying images. A copy of the written consent is available for review by the Editor-in-Chief of this journal on request.

## Ethical approval

Ethical Approval was waived by the authors institution.

## Sources of funding

None.

## Author contribution

All authors contributed to the study conception and design. Material preparation, data collection, and analysis were performed by Sakiko Soutome, Saki Hayashida, Souichi Yanamoto, Kota Morishita, and Masahiro Umeda. The first draft of the manuscript was written by Mitsunobu Otsuru, and all authors commented on the previous versions of the manuscript. All authors read and approved the final manuscript.

## Guarantor

Mitsunobu Otsuru.

## Research registration

None.

## Declaration of competing interest

None.
